# A Journey Through Time – Study Protocol for a Randomized Controlled Trial Testing the Add-on Effects of Imagery Rescripting to Ongoing Cognitive Behavioural Therapy in Patients With Depressive Disorders

**DOI:** 10.32872/cpe.16709

**Published:** 2025-11-28

**Authors:** Amelie Endres, Anja Schaich, Arnoud Arntz, Eva Fassbinder, Fritz Renner

**Affiliations:** 1 Department of Clinical Psychology and Psychotherapy, Institute of Psychology, University of Freiburg, Freiburg, Germany; 2 Department of Psychiatry and Psychotherapy, University of Lübeck, Lübeck, Germany; 3 Department of Clinical Psychology, University of Amsterdam, Amsterdam, The Netherlands; 4 Clinic for Psychiatry, University of Kiel, Kiel, Germany; University of Lausanne, Lausanne, Switzerland

**Keywords:** imagery rescripting, major depressive disorder (MDD), distressing memories

## Abstract

**Background:**

Patients with depression often report recurring memories of stressful events from the past (e.g. experiences of rejection, emotional or physical abuse). These distressing memories, commonly dating back to childhood, can contribute to the development and maintenance of depression through their impact on cognitive schemas. The method of *imagery rescripting (ImRs)* addresses distressing memories and the associated emotions directly: With therapeutic support, patients recall the respective memory and modify the memory during imagination in such a way that the emotional quality and meaning of the memory changes. In this randomized trial, we will assess the impact of a three-session ImRs intervention within standard cognitive behaviour therapy (CBT) for depression, comparing it to an active control intervention (imagery relaxation).

**Method:**

Sixty-six patients with MDD who are currently receiving treatment (CBT) will be randomized to either (1) the experimental condition (ImRs) or (2) the control condition (imagery relaxation). Reduction of depressive symptoms, measured by the Beck Depression Inventory (BDI-II) is the primary outcome. The BDI-II will be assessed at baseline, post-intervention, and at 4-week and 8-week follow-ups.

**Discussion:**

This study will help to clarify whether adding three ImRs sessions improves the effectiveness of CBT for depression. We outline the next steps for future research and highlight the potential of this novel intervention for depression.

## Highlights

- Most patients with depression report distressing memories, making them a key treatment target.
- Imagery Rescripting (ImRs) may enhance CBT by reducing negative emotions tied to distressing memories.
- This RCT tests ImRs as an add-on to CBT for depression.

Depression, characterized by low mood and diminished interest in previously rewarding activities, is a pervasive mood disorder and a global health concern (American Psychiatric Association, 2013; Ferrari et al., 2013; James et al., 2018; Whiteford et al., 2013). Despite the effectiveness of established psychological treatments like Cognitive Behavioural Therapy (CBT; Beck, 1970) in alleviating depressive symptoms, approximately 40% of patients do not respond adequately, underscoring the need for innovative interventions (Cuijpers et al., 2021; Zhdanava et al., 2021). The gap between the number of patients in treatment and treatment success points to the importance of further improving the treatment of depression.

A crucial aspect often overlooked in current psychological treatments is the presence of distressing memories, reported by about 80% of depressed patients (Brewin et al., 1996; Newby & Moulds, 2011a; Payne et al., 2019). These memories, even in the absence of traumatic events, predict depressive symptoms and persist to trouble patients who have recovered from depression (Buckman et al., 2018; Kuzminskaite et al., 2022; Nelson et al., 2017; Newby & Moulds, 2011b; Payne et al., 2019). Thus, distressing memories are not only common in depression but could also be a significant treatment target. Distressing memories are associated with unpleasant emotions such as sadness, shame, fear, or anger. While traditional CBT addresses cognitive and behavioural aspects of emotional problems, recent psychotherapy research underscores the importance of bringing the patient’s emotions into focus (Greenberg, 2010, 2017; Peluso & Freund, 2018). One technique to address both the distressing memories and the associated negative emotions is Imagery Rescripting (ImRs; Arntz & Weertman, 1999). ImRs, a therapeutic technique integrated into therapeutic approaches or used as a standalone intervention (Kip et al., 2023), addresses distressing memories in a two-step process (Arntz & Weertman, 1999). First, the individual vividly imagines the distressing memory, engaging various sensory modalities and experiencing the connected emotions. In the second step, the sequence of events is imaginatively transformed into a more desirable direction. This is achieved through the introduction of a supportive figure – such as the therapist or the patient’s adult self – who addresses the individual’s emotional needs. ImRs does not alter original memory content but aims to reduce the memory’s emotional meaning and emotional impact (Aleksic et al., 2024; Arntz, 2012; Arntz & Weertman, 1999). Arntz (2012) hypothesizes that the central mechanism of change in ImRs is the modification of maladaptive schemas, with emotional changes occurring as a by-product of this process. This perspective emphasizes the cognitive restructuring aspect of ImRs, where the reimagining of events leads to a shift in underlying belief systems, subsequently influencing emotional responses. Experimental research supports the idea that mental imagery has a profound impact on emotion (Holmes et al., 2008, 2009; Ji et al., 2016) and that ImRs reduces negative emotions and distress associated with negative memories (Çili & Stopa, 2021; Nilsson et al., 2012; Reimer & Moscovitch, 2015; Strohm et al., 2021). The positive impact of ImRs on negative emotions as well as on the distressing memories seems promising for the treatment of depression.

Despite its early recognition in CBT (Beck, 1970), the clinical applications of mental imagery within a CBT framework were relatively under-researched until recently, with pilot studies of ImRs as standalone interventions for depression demonstrating significant effects on symptom severity (Brewin et al., 2009; Ma & Lo, 2022; Pile et al., 2021; Renner & Holmes, 2018). Additionally, research has shown that engaging in mental imagery of future positive events can increase behavioural activation in individuals with major depressive disorder, further highlighting the potential of imagery-based interventions in depression treatment (Renner et al., 2017). Meta-analyses by Kip et al. (2023) and by Kroener et al. (2023) have further supported the efficacy of ImRs in treating various mental disorders associated with aversive memories, including depression, revealing large pre-post effect sizes across different disorders and highlighting its potential as a transdiagnostic intervention. Building on this evidence, a recent controlled pilot study by Kanczok et al. (2024) investigated the combined use of cognitive restructuring (CR) and ImRs compared to treatment as usual among inpatients with moderate and severe depression. The study found that patients in the intervention group (receiving CR and ImRs) achieved significantly greater improvements in depressive symptoms over time compared to the treatment-as-usual group. While these findings are promising, further research is needed to investigate the specific additional effect of using ImRs during regular cognitive behavioural therapy, particularly in outpatient settings and with larger sample sizes.

In addition to the open research questions regarding the extent to which ImRs affects depressive symptoms, there is also a lack of evidence regarding the potential mechanisms that play a role in ImRs. Studies have shown some evidence for different potential mechanisms on a cognitive level that might be responsible for the effect of ImRs: One aspect that several studies point out is the positive impact of ImRs on different aspects of a person’s self-representation (Lee & Kwon, 2013; Wild et al., 2008), self-belief (Cooper, 2011) and self-esteem (Çili et al., 2017). There are study results that indicate that the negative core beliefs associated with the distressing memories reduce in subjects treated with ImRs (Reimer & Moscovitch, 2015). Mancini and Mancini (2018) suggest that the effect of ImRs is based on a reduction of the ‘meta-emotional problem’, which is linked to a better acceptance of the negative emotions associated with the distressing memories. In the treatment of posttraumatic stress disorder (PTSD) related to traumas experienced in childhood, changes in strengths of encapsulated beliefs and distress of the index trauma preceded changes of PTSD-severity in ImRs (Rameckers et al., 2024), supporting the theory that change in emotional meaning of the memory underlies the effects of ImRs. Assmann et al. (2024) investigated the role of cognitions in the treatment of childhood-related PTSD using ImRs. Their study found that changes in trauma-related cognitions significantly mediated the relationship between ImRs and reductions in PTSD symptoms. These results highlight the critical role of cognitive processes in the effectiveness of ImRs and suggest that targeting maladaptive cognitions may be a key mechanism through which ImRs facilitates therapeutic change in PTSD. Further research is needed on the effects of ImRs on different aspects of cognition and emotion as well as on the ‘meta-emotional problem’.

Current research points to the problem that the effect of ImRs on depression has not been tested sufficiently in studies yet, especially not as an adjunct to regular cognitive behavioural therapy. Furthermore, the mechanisms of action of ImRs for depression remain unclear. This randomized controlled trial aims to expand the research on ImRs in depression. The primary objective is to evaluate the effectiveness of ImRs as an add-on to standard CBT for major depressive disorder (MDD). Patients receiving CBT in routine care are randomized to receive either three sessions of ImRs or an active control condition (imagery relaxation). We hypothesize that participants in the ImRs condition will show greater reductions in depressive symptoms from pre-intervention to post-intervention and follow-up. Although ImRs may be applicable across a range of disorders (Kip et al., 2023), we chose to focus specifically on depression in the present study for several reasons: First, the high prevalence of distressing memories among individuals with depression suggests a particularly strong clinical relevance of ImRs in this context (Brewin et al., 1996; Newby & Moulds, 2011a; Payne et al., 2019). Second, existing studies have primarily evaluated ImRs as a standalone intervention; its potential additive effect when embedded within standard CBT for depression remains largely unexplored. Third, depression continues to be among the most prevalent and burdensome mental health conditions, underlining the need for innovative adjunctive treatments. Given these considerations, we see depression as a particularly meaningful starting point for examining the clinical utility of ImRs. Importantly, comorbidities are not excluded in our study, reflecting the complex presentations often encountered in routine clinical practice. Secondary objectives of this trial include examining changes in associated psychological variables (e.g., repetitive negative thinking, emotional capabilities, self-compassion, self-esteem). In addition, exploratory analyses will investigate potential mechanisms underlying the effects of ImRs.

## Method

### Design

The study is a two-arm randomized controlled trial with one active intervention condition (Imagery Rescripting, ImRs) and one active control condition (imagery relaxation). Participants in both conditions are invited to complete questionnaires at baseline session (Session 1), Weeks 1-3 after baseline (intervention Sessions 1-3) and at post and Follow-up 1 and 2 (Week 1, 4 and 8 after the last session). The medical ethics committee of the University of Freiburg has approved the study (Approval number: 22-1518-S2). The study is pre-registered at the German Clinical Trials Register https://drks.de/search/de/trial/DRKS00031495.

### Participants

Inclusion criteria are (a) meeting the diagnostic criteria for a current major depressive disorder (MDD), as assessed with a SCID-5-CV interview (patients with comorbid disorders are eligible to participate), (b) age 18 years or older, (c) sufficient proficiency of German language to complete questionnaires. Patients are excluded from participation if they are at high risk of suicidality, defined as the presence of acute suicidal ideation with concrete plans or intent. This is assessed during the standard intake procedure at the outpatient clinic where the study is conducted. Diagnostic information is based on the initial clinical interview (SCID) conducted by the therapist at the outpatient clinic; no additional study-specific diagnostic assessment will be conducted. Participants are informed about the study at the beginning of their treatment but may enrol at any point during their ongoing CBT. The number of standard CBT sessions completed at study entry will be documented and reported. It is important to note that CBT is delivered as part of routine care in the outpatient clinic and is not part of the experimental study protocol. No efforts are made to standardize or influence the content, structure, or dose of the ongoing CBT. However, information on the number of CBT sessions and the use of imagery techniques during therapy will be collected post-hoc via therapist questionnaires.

### Sample Size

Previous research has demonstrated the effectiveness of Imagery Rescripting (ImRs) in treating various mental disorders, with large effect sizes reported when compared to inactive control conditions (Morina et al., 2017). Ma and Lo (2022) conducted a randomized controlled trial comparing ImRs to cognitive restructuring (CR) in depression treatment. Their findings revealed that both ImRs and CR led to significant reductions in depressive symptoms, with medium to large effect sizes observed. In the present study, we aim to build on these findings by comparing ImRs to an active control condition (relaxation) in the context of ongoing Cognitive Behavioural Therapy (CBT) for depression. Since both study arms receive standard CBT and the control condition is active rather than passive, we expect smaller between-group differences than in previous trials. Specifically, we anticipate moderate differences in the reduction of depressive symptoms between the two interventions. To determine the required sample size, we conducted a simulation-based power analysis using the simr package in R. The analysis was based on a linear mixed-effects model that reflects the longitudinal structure of the study, including four repeated measurement points and a random intercept for each participant. Assuming a moderate group × time interaction effect (β = 0.25) at the final follow-up (FU2), the simulation indicated that a total sample size of approximately 60–70 participants would yield a statistical power of 85–90%. To account for potential dropouts while keeping the study feasible in terms of recruitment and implementation effort, we planned a total sample size of 66 participants with equal allocation to both conditions. This sample size is expected to be sufficient to detect moderate effects over time.

### Recruitment

Eligible potential participants will receive written study information from their therapist in the outpatient clinic. If they are interested in participating, they are asked to return a consent form to their therapist, giving permission to be contacted by the research team. A member of the research team will then contact the patient to discuss the study, clarify any questions and see if the patient is interested in participating in the study. If interested, an appointment for the initial session (introductory session) will be scheduled. Informed consent for study participation will be signed at the introductory session.

### Therapists

Study therapists will be four graduated psychologists in an advanced stage of clinical training to become fully licensed psychotherapists who are working at the outpatient treatment center of the University of Freiburg. Before the start of the study, study therapists received 15 hours of training in the ImRs procedure by Arnoud Arntz and training in the imagery relaxation procedure by Fritz Renner. Clinical supervision meetings are planned on a regular basis throughout the study.

### Introduction Session (Baseline)

Prior to the start of the study’s therapeutic intervention, participants will complete an online baseline assessment. Subsequently, an initial 60-minute introductory session will be scheduled with each participant. In the introductory session, the participants will get to know their study therapist and distressing memories will be identified using a semi-structured interview based on the work of previous studies (Ma & Lo, 2022; Patel et al., 2007). There will be no restriction on the timeframe where the memory originated as previous work suggests that ImRs can effectively be applied to “older” childhood memories as well as more recent memories. Participants will be asked to recall three distressing memories and will be asked about the content of each memory, their age at the time when the event occurred and the context in which the event occurred. Core beliefs associated with the memory will be assessed by using a standardized protocol. The specific emotions and the current distress caused by the core beliefs will be assessed as well.

### Randomization

After the introduction session, patients will be randomized into one of the two conditions using a computer script performing block randomization (1:1, block size = 6). Randomization will be done by a researcher who is not involved in the study sessions. After the randomization, the patients will be sent a video explanation in which their study therapist explains what is going to happen in the respective condition. To ensure consistency and standardization in how each condition is introduced, participants receive a brief video following randomization in which their assigned study therapist explains the rationale, structure, and expectations of the respective intervention. These videos aim to enhance transparency, facilitate engagement, and reduce time needed for explanation during the first session, thereby allowing more time for therapeutic work.

### Duration of the Study

**Figure 1:**
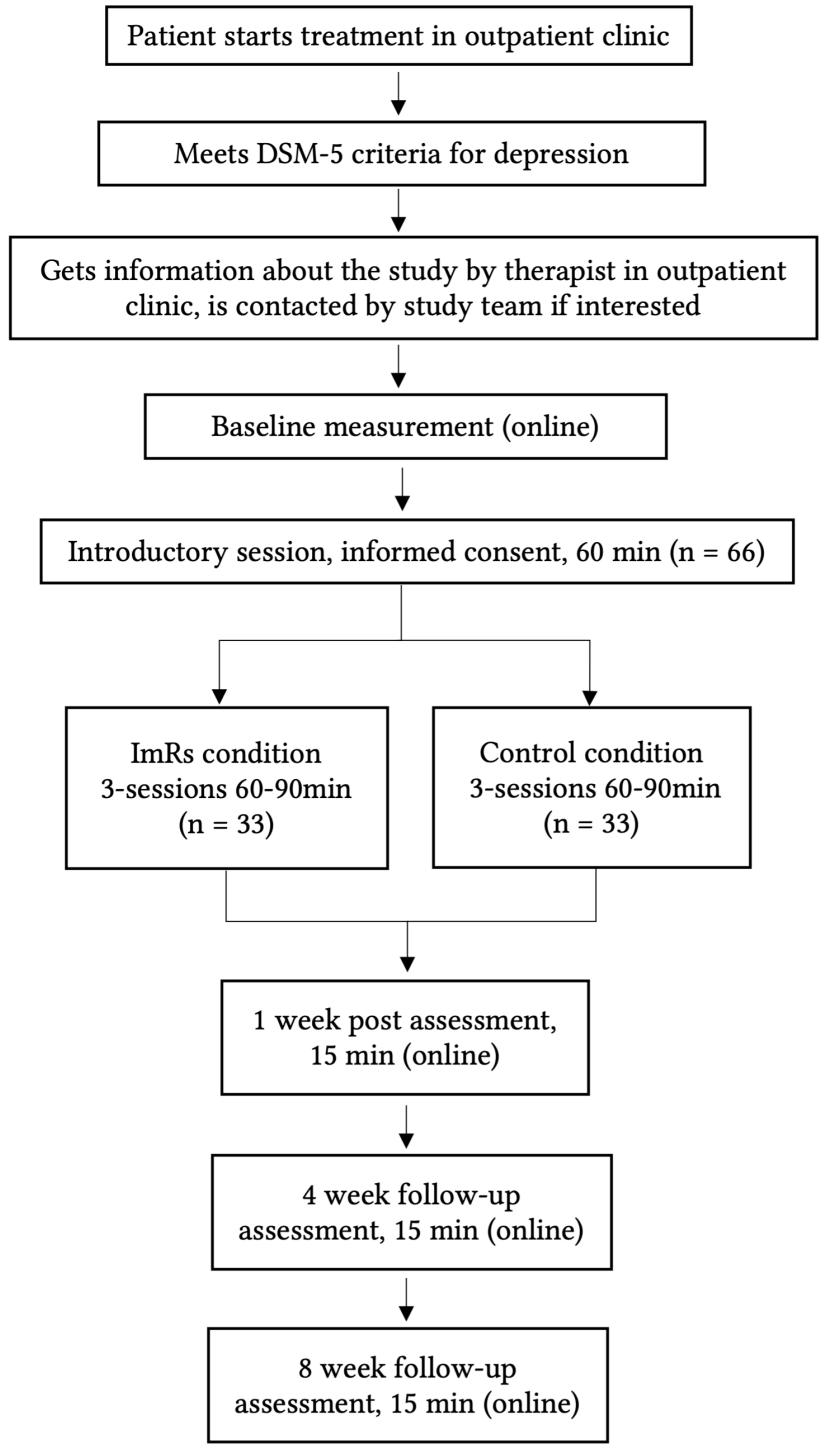
Study Procedure

The three intervention sessions will take place weekly so the duration of the study therapy sessions will extend over four weeks (see Figure 1). Eight weeks after the last study therapy session, the last follow-up measurement will take place (online). Longer follow-up intervals (e.g., at three or six months) were not implemented in this trial due to feasibility constraints and methodological considerations. Since participants continue to receive non-standardized CBT throughout and beyond the follow-up period, longer intervals would likely introduce greater variability in treatment exposure, making it more difficult to attribute outcomes specifically to the experimental intervention. Shorter follow-up intervals were therefore chosen to maintain a clearer link between the experimental intervention and outcome assessments. The duration of the sessions in both experimental and control conditions is comparable (60 – 90 minutes) to control for time and therapist contact. The study procedure is also presented in Figure 1. Participants who take part in all sessions of the study will receive €50 as compensation for their time investments.

### Measures

#### Primary Outcome

Changes in Depressive Symptom severity, assessed by the Beck Depression Inventory-II (BDI-II; Beck et al., 1996) will be the primary outcome. The BDI-II is a 21-item self-report instrument assessing depressive symptoms. The items are rated from 0 to 3, with 3 for the most depressed mood. A score 0 – 13 indicates minimal depression, 14 – 19 mild depression, 20 – 28 moderate depression and 29 – 63 severe depression. The BDI-II will be measured before randomization (baseline), one week after the last session (post-intervention) and 4 weeks and 8 weeks after the last session (follow-up). The BDI-II was chosen as the primary outcome due to its strong psychometric properties, sensitivity to change, and feasibility for repeated online assessments, thereby minimizing participant burden and facilitating efficient data collection.

#### Secondary Outcomes

We will use the following measures at every measurement (before randomization (baseline), just prior to every session, one week after the last session (post-intervention) and four weeks and eight weeks after the last session (Follow-up 1 and 2)): Depressive symptom severity, measured by the depression module of the Patient Health Questionnaire (PHQ-9; Kroenke et al., 2001); Rating (0 – 100) of the belief that the core beliefs captured in the baseline session are true; Rating (0 – 100) of the emotions that are associated with the memories and core beliefs captured in the baseline session; Rating (0 – 100) of the burden of the memories for the patient. The PHQ-9 was included alongside the BDI-II to allow for convergent validation of depressive symptom trajectories. In addition, due to its brevity and ease of administration, the PHQ-9 is used at each study session, making it possible to assess depressive symptoms at more frequent time points than with the BDI-II. This enables a more fine-grained analysis of symptom changes across the intervention period.

The following measures will be administered before randomization (baseline), one week after the last session (post-intervention) and four weeks and eight weeks after the last session (Follow-up 1 and 2): *Repetitive negative thinking*, measured by the Perseverative Thinking Questionnaire (PTQ; Ehring et al., 2011); *Emotional capabilities*, measured by the Self-Assessment of Emotional Capabilities (SEK-27; Berking & Znoj, 2008); *Self-compassion*, measured by the short version of the German Self-Compassion Scale (SVS-SV; Hupfeld & Ruffieux, 2011); *Self-efficacy*, measured by the German global self-efficacy expectancy scale (SWE; Jerusalem & Schwarzer, 1999); *Beliefs about emotions*, measured by the German version of the Emotion Beliefs Questionnaire (EBQ; Grüning et al., 2021); *Self-esteem*, measured by the German version of the Rosenberg Self-Esteem Scale (RSES; von Collani & Herzberg, 2003). At post-intervention, we will measure *satisfaction with treatment*, measured by the German patient satisfaction questionnaire (ZUF-8; Schmidt et al., 1989) adapted for the study as well as the *working alliance* between patient and study therapist, measured by the working alliance inventory (WAI; Wilmers et al., 2008). At the post-intervention assessment, the patients will also be asked in an open-response format, to what extent they think they have benefited from the study.

#### Additional Measures

To evaluate possible confounding variables, the following constructs are measured: *Childhood Trauma*, measured by the Childhood Trauma Questionnaire (CTQ; Bernstein & Fink, 1998), which will be administered during the baseline measurement as ImRs interventions were originally developed for intrusive memories following traumatic events; *Time of intervention*: Time interval (in days) between the participants’ regular CBT session and the study therapy session for each of the three intervention sessions; *Comorbid disorders*, as measured with the SCID-5-CV interview at the outpatient clinic before the start of the study;

*Therapeutic techniques* used in patients’ routine therapy, as well as the *amount of regular therapy sessions* received during the study period, will be recorded via a questionnaire sent to the treating therapists at the outpatient clinic after the patient has completed the final follow-up assessment (FU2). The CBT conducted alongside the study intervention is part of routine clinical care and not part of the study protocol. It follows a naturalistic format without a standardized manual, predefined number of sessions, or fixed structure. Consequently, the dose and content of CBT may vary between participants. To monitor potential overlap with imagery-based methods, the therapist questionnaire includes items assessing whether, and to what extent, imagery techniques or imagery rescripting were used during the course of therapy.

#### Manipulation Check

To check if patients in both conditions engaged in mental imagery, they will be asked to note how vivid the imagery was: After each imagery exercise in the ImRs condition, the patient will be asked to note how vivid the imagery of the respective situation was on a scale from 1 (not vivid at all) to 10 (extremely vivid). To check if the session contained any deviations from protocol, the study therapists will be asked to report any deviations from protocol after each session. To ensure that the conditions are executed as intended, all sessions will be recorded on video and evaluated by two independent raters for adherence to the protocol (adherence scales for both interventions were worked out by the study group).

### Experimental Condition: ImRs

The ImRs intervention is based on previous experimental ImRs protocols (Arntz & Weertman, 1999; Ma & Lo, 2022). The relaxation intervention in the control group is based on standardized relaxation protocols (Toussaint et al., 2021).

#### ImRs Session 1 and 2

During ImRs intervention Sessions 1 and 2, participants will be asked to choose one of the three distressing memories. Then they will be asked to delve into the distressing (childhood) memory in their imagination. They will be asked to give a verbal narrative of the contextual and sensory details of the event by questions as for example “what do you see?”, “what do you hear?” and “who else is there?”, as well as to experience and report the emotions that are activated, to share thoughts that go through their mind, and to express what they emotionally need. When the participants’ emotions are sufficiently activated and the most difficult part of the memory is imagined, the therapist will step into the image and change the outcome to a positive ending. The therapist assists the younger self of the participant in addressing the distressing situation, which may include confronting a perpetrator if present. The therapist helps in defending and supporting the younger self of the patient. The therapist will then take care of all other emotional needs of the younger self, helping to process associated emotions and reduce the emotional distress linked to the event. This may involve introducing a sense of safety or control, and when applicable, confronting any responsible parties. Examples of interventions include providing comfort, offering protection, or empowering the younger self. The therapist continues to support the child or younger self of the participant until feeling safe and having all emotional needs satisfied.

#### ImRs Session 3

During ImRs Intervention Session 3, participants will delve into their remaining (childhood) memory in their image and will be again asked to give a verbal narrative of the contextual and sensory details of the event, as well as to share the emotions, thoughts and needs that are activated. At the most difficult moment the patient is instructed to change perspective and imagine to enter the scene as their present adult self and help their younger self. As modelled in the two sessions by the therapist, the adult self should now intervene in the situation. This may involve addressing a perpetrator and taking care of the needs of the younger self. In the final phase, participants will be asked to once again alter their perspective, this time re-entering the scene as their younger self to experience the support and intervention of their adult self. The younger self is also encouraged to request additional interventions as needed.

#### Possible Variations of the Sessions

If the participants want to rescript a distressing memory that was not explored in the introductory session, they will be allowed to use that new situation. In that case, the therapist will briefly assess the new memory using the same structure as in the introductory session (content, age, context, core beliefs, emotional impact), ensuring continuity and consistency of the intervention. If participants do not wish or feel able to personally assist their younger selves in the third session, the session can be conducted as in Sessions 1 and 2.

### Control Condition: Mental Imagery Relaxation Condition

The intervention in the control condition will be an imagery-based relaxation exercise based on established protocols (Toussaint et al., 2021). As in the experimental condition, there will be three experimental sessions lasting between 60 – 90 minutes. Guided imagery relaxation is an established technique for stress reduction. Its effectiveness in reducing symptoms of depression, anxiety, and stress has been demonstrated in several studies (Apóstolo & Kolcaba, 2009; Beizaee et al., 2018; Costa & Barnhofer, 2016). The intervention periods in these studies ranged from one week to four weeks, indicating its potential to produce measurable effects within a relatively short time frame. During this intervention positive imagery is used to invoke sensory experiences and physiological responses. Participants will be given the choice between different topics (e.g. a beach, a forest, a lake, a sky full of stars). Then they will be instructed to sit down in a comfortable manner and to imagine themselves in the scene. The therapist will read out a text about the scenery (e.g. how does the sand under their feet feel). The imagery will be combined with breathing exercises. If time permits participants might complete several relaxation imagery exercises during one session with short intermediate breaks.

### Data Analyses

To address the longitudinal nature of our data and potential missing values, we will employ a multilevel approach. This method allows us to model individual trajectories of change over time while accounting for between-person differences. The multilevel model will include time as a within-subject factor and condition as a between-subject factor. The interaction effect between time and condition is of main interest, as it will indicate whether the rate of change in depressive symptoms differs between the intervention and control group. All primary analyses will be conducted using an intention-to-treat (ITT) approach, including all randomized participants. Missing data will be handled using mixed-effects models, which are robust to missingness under the assumption of missing at random. Analysis will be conducted in R (R Core Team, 2025). In case of substantial differences between conditions in demographic or any of the potentially confounding variables listed previously (e.g., number of CBT sessions before intervention), the variable(s) will be added as covariates to the model. For the analysis of secondary outcomes (e.g., repetitive negative thinking, emotional capabilities, self-compassion, self-esteem), we will employ a similar multilevel modelling approach. Each secondary outcome will be analysed separately, with time and condition as predictors, and the time-by-condition interaction as the primary effect of interest. Given the number of secondary outcomes, we will apply a correction for multiple testing to mitigate the risk of Type I errors across models. Effect sizes will be reported alongside *p*-values to aid interpretation. Results will be treated as exploratory, and full model outputs (including corrected and uncorrected *p*-values) will be provided in the supplementary material to ensure transparency. This will allow us to examine how these outcomes change over the course of the study and whether these changes differ between the ImRs and control conditions.

### Exploratory Analyses

We will present the drop-out rate alongside descriptive statistics and include qualitative feedback from participants regarding their experiences with the study. To investigate potential mechanisms of the effects of ImRs, we will conduct mediation analyses within the multilevel framework. Specifically, we will explore whether changes in secondary outcomes mediate the relationship between the intervention condition and changes in depressive symptoms.

## Discussion

We presented a study protocol for a randomized controlled trial testing the additional effect of ImRs on depressive symptoms in individuals who are already in treatment (CBT). We hypothesized that, compared to an active control condition, the participants receiving ImRs would have a greater reduction of depressive symptoms.

While there are pilot studies showing significant effects of ImRs on depressive symptom severity (Brewin et al., 2009; Ma & Lo, 2022), this study aims to replicate and extend these promising results. Importantly, no studies have yet examined the additional effect of ImRs during regular CBT for depression. By investigating potential synergistic effects of ImRs and CBT, this study could provide valuable insights into enhancing treatment outcomes for depression. If participants in the ImRs condition show greater improvements in depressive symptoms, it would suggest a promising avenue for augmenting existing therapies. This is particularly significant given that current treatment options for depression are often insufficient for many patients (Cuijpers et al., 2021). Another important aspect that this study attempts to clarify is the underlying mechanisms that may be involved in the effect of the ImRs technique on depressive symptoms. This study contributes to the research on potential mechanisms by assessing the change of different psychological variables during the course of the study and by analysing exploratorily whether changes in psychological variables mediate the effect of ImRs on depressive symptoms.

One potential limitation of the study is that the therapists who conduct the experimental interventions of this study are not the same therapists who conduct the ongoing regular CBT. The ImRs and relaxation interventions are therefore not integrated into the ongoing treatment. Moreover, competence and experience levels of the therapists providing the ongoing regular CBT varies. While this approach enhances the ecological validity of our research, it introduces an analytical challenge. Because many therapists are involved in the regular therapy, and each therapist likely sees only few study participants, we can not effectively account for how individual therapists might influence the results. We do not have enough participants grouped with each therapist to reliably measure the impact that specific therapists might have on the outcomes. One other limitation is that the study is powered to find medium to large effects but is underpowered to find small or medium effects between the two active interventions. Furthermore, the effect size assumptions underlying the power calculation may be overestimated, as they are based on studies conducted without concurrent CBT, whereas in the present study, the intervention is delivered in parallel to ongoing psychotherapy. This parallel treatment setting may reduce the observable effect sizes and should be considered when interpreting the results. Finally, this study exclusively relies on self-report measures for primary and secondary outcomes. While this approach allows repeated, low-burden assessment, clinician-rated interviews could provide additional information on how many patients meet diagnostic criteria following the intervention. Additionally, we acknowledge that some participants might no longer fulfil the criteria for a Major Depressive Disorder diagnosis at the time of study inclusion, despite initially meeting them during screening. This should be considered when interpreting the results, as it may influence the generalizability and observed treatment effects.

Overall, we aim to expand the research on innovative interventions for depressive disorders with this study. The results will contribute to a better understanding of the effects of ImRs on depression, as well as its potential added value when delivered alongside ongoing cognitive behavioural therapy. Given that the primary outcome is assessed before the completion of CBT, interpretations regarding additive effects should be considered exploratory.

## Supplementary Materials

The Supplementary Materials contain the preregistration for the study (see Endres et al., 2023S).



EndresA.
SchaichA.
ArntzA.
FassbinderE.
RennerF.
 (2023S). Journey through time – Imagery rescripting of distressing memories
[Preregistration; Registration No.: DRKS00031495]. PsychOpen. https://drks.de/search/de/trial/DRKS00031495


## Data Availability

Upon completion of recruitment and data analysis, the data of the presented study will be made available via the Open Science Framework. Alternatively, data will be made available by the authors on reasonable request.

## Reporting Guidelines

This study was reported following the CONSORT 2010 Statement (Schulz et al., 2010).

## Preregistration

The study is registered at the German Clinical Trials Register (DRKS; registration number: DRKS00031495).
